# Evaluation of Three Phenotypic Methods for Detecting Metallo-β-Lactamase in Clinical Isolates of Acinetobacter baumannii

**DOI:** 10.7759/cureus.95031

**Published:** 2025-10-21

**Authors:** Prashanth K Guddeti, Bhawani S Verma, Ramanath Karicheri, Md Abdullah, Harshada Shah

**Affiliations:** 1 Department of Microbiology, Dr. Ulhas Patil Medical College & Hospital, Jalgaon, IND; 2 Department of Microbiology, Index Medical College Hospital and Research Centre, Indore, IND

**Keywords:** carbapenem-resistant acinetobacter baumannii, double-disk synergy test (ddst), mbl e-test, metallo-beta-lactamase (mbl), modified hodge test (mht)

## Abstract

Background

Carbapenem resistance mediated by metallo-β-lactamase (MBL) production in *Acinetobacter baumannii* represents a growing global health threat. Early detection of MBL-producing organisms is essential for the prompt administration of appropriate therapy to effectively manage infections.

Materials and methods

This cross-sectional study was conducted in the Department of Microbiology, Index Medical College Hospital and Research Centre (IMCHRC), Indore, from October 2019 to September 2021. *A. baumannii* strains showing resistance or intermediate susceptibility to imipenem and meropenem were subjected to MBL detection using phenotypic methods, namely the Modified Hodge Test (MHT), Double-Disk Synergy Test (DDST), and MBL E-test.

Results

Out of 143 clinical isolates of *A. baumannii*, 132 isolates were resistant to carbapenems across various clinical specimens. Among these 132 carbapenem-resistant *A. baumannii* (CRAB) isolates, MBL production was detected in 132 (100%) by the E-test, 126 (95.45%) by MHT, and 120 (90.9%) by DDST. Six isolates (4.54%) were negative by MHT, and 12 isolates (9.1%) were negative by DDST. Sensitivity and specificity of the phenotypic methods were analyzed using the MBL E-test as the gold standard for detecting MBL production in CRAB isolates.

Conclusions

Clinical isolates of *A. baumannii* producing MBL should be routinely screened in diagnostic laboratories to help control resistance and guide therapy, as MBL production is associated with multidrug resistance and confers resistance to carbapenems, which are reserved for severe infections. Among the phenotypic methods, the MBL E-test demonstrated superior accuracy compared to MHT and DDST for detecting MBL-producing *A. baumannii* isolates.

## Introduction

Carbapenem resistance due to metallo-β-lactamase (MBL)-producing *Acinetobacter baumannii* is an emerging global threat [1]. Over the past two decades, *A. baumannii* isolates from various infections have increasingly exhibited resistance to β-lactam antibiotics, particularly cephalosporins and carbapenems, posing a significant worldwide challenge [2]. Multiple mechanisms, including enzyme-mediated resistance, genetic mutations, efflux pumps, porin alterations, and changes in outer membrane protein components, contribute to reduced therapeutic efficacy in nosocomial infections caused by *A. baumannii*.

Carbapenems are β-lactam antimicrobials, and enzyme-mediated resistance arises when bacteria produce β-lactamases that inactivate these agents [3]. In *A. baumannii*, carbapenem resistance is predominantly due to class D β-lactamases, commonly known as OXA-type carbapenemases [4-6]. β-lactamases are historically categorized into four classes (A, B, C, and D) according to Ambler’s classification system: classes A, C, and D are serine β-lactamases, whereas class B consists of MBLs [6-8].

MBLs hydrolyze all β-lactam antibiotics, including carbapenems, except aztreonam (a monobactam). Strains producing MBLs are not susceptible to serine β-lactamase inhibitors, such as clavulanate [9,10]. Early detection of carbapenemase production is critical for implementing effective infection control measures and preventing the spread of resistant strains. For initial screening, methods such as the E-test for MIC determination, disc diffusion, and automated antimicrobial susceptibility systems are commonly used. Phenotypic confirmation techniques include the Modified Hodge Test (MHT), EDTA-impregnated disc test, 2-mercaptopropionic acid disc test, boric acid disc test, and microdilution assays [11,12].

Although PCR genotyping has long been considered the gold standard for detecting MBL genes, its high cost limits routine use, and it is typically reserved for research purposes [1,13]. Consequently, most diagnostic laboratories continue to rely primarily on culture-based phenotypic tests to rapidly detect MBL activity. Early identification of MBL-producing organisms is essential for the timely administration of appropriate therapy to effectively manage infections [13]. Given the limited availability of molecular methods in many laboratories, this study aimed to evaluate and compare three phenotypic techniques for detecting MBL-producing *A. baumannii*.

## Materials and methods

The present cross-sectional study was conducted from October 2019 to September 2021 in the Department of Microbiology, Index Medical College Hospital and Research Centre, Indore. A total of 168 consecutive, nonduplicate clinical isolates of *Acinetobacter *were obtained from various clinical samples of hospitalized patients who provided written informed consent. All *Acinetobacter *species were isolated using standard microbiological techniques [10,14-16]. Antimicrobial susceptibility testing was performed using the disk-diffusion method on Mueller-Hinton agar (MHA; HiMedia Laboratories Private Limited, Mumbai, India), and results were interpreted according to the Clinical and Laboratory Standards Institute (CLSI) 2019 guidelines [17].

Detection of MBL production

*A. baumannii* strains showing resistance or intermediate susceptibility to imipenem (IPM) and meropenem (MRP) were subjected to MBL detection using phenotypic methods, namely the MHT, Double-Disk Synergy Test (DDST), and MBL E-test.

MHT

**Figure 1:**
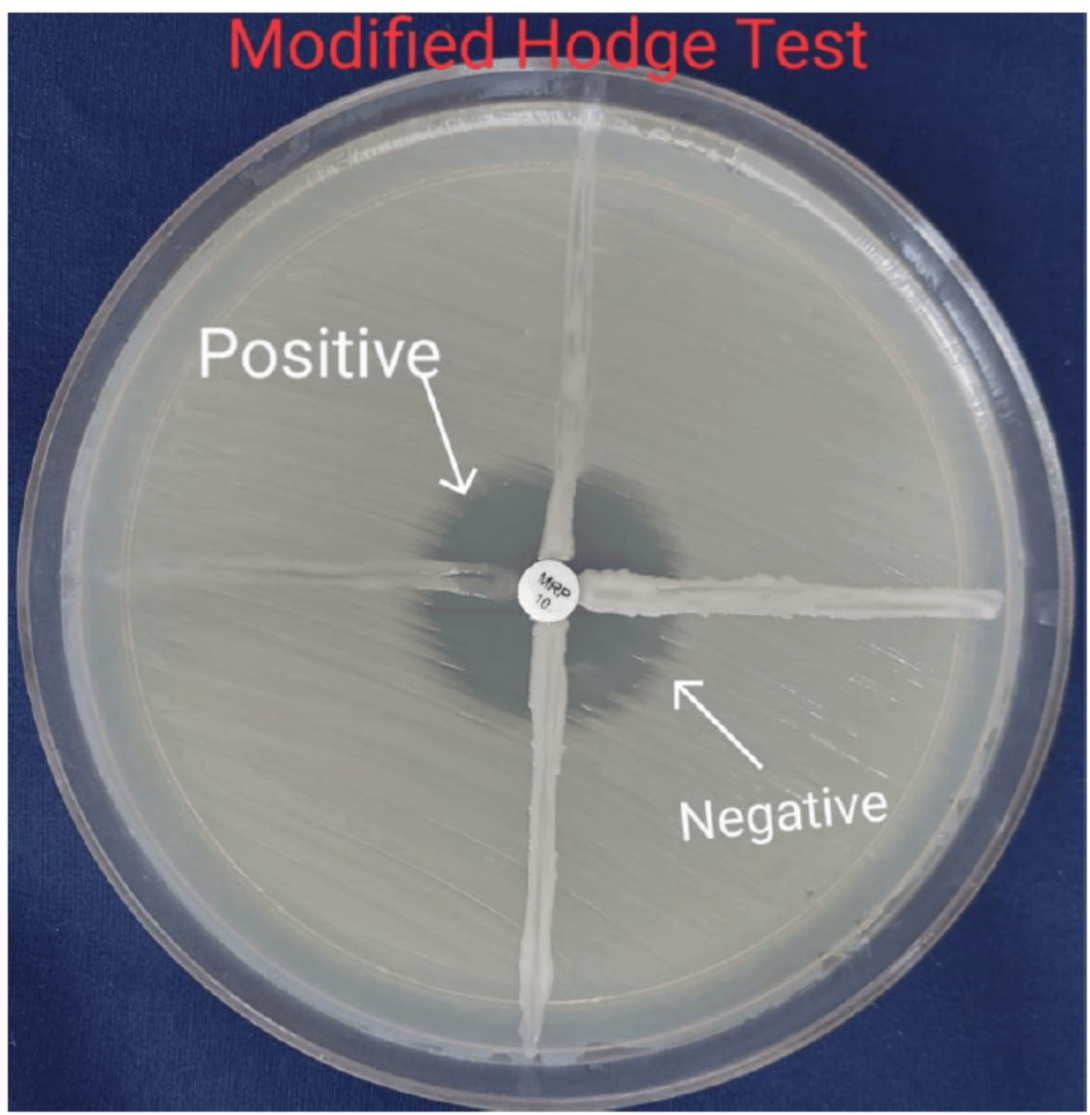
MHT for the detection of MBL producers Positive results show a distorted, cloverleaf-shaped zone of inhibition, whereas negative results lack this characteristic distortion, as illustrated in the figure. MBL, metallo-β-lactamase; MHT, Modified Hodge Test

*Escherichia coli* ATCC 25922 (an indicator organism susceptible to carbapenems) was cultured in peptone water to a 0.5 McFarland standard and lawn-cultured onto an MHA plate using a sterile cotton swab. After the plate dried, a 10 µg IPM disc was placed in the center of the lawn culture. Test isolates of *A. baumannii, *along with positive and negative control strains, were streaked heavily from the edge of the IPM disc outward to the plate periphery in four directions. Plates were incubated overnight at 37°C. A positive result was indicated by a distorted, cloverleaf-shaped zone of inhibition, whereas a negative result showed no cloverleaf-shaped distortion (Figure 1) [18,19].

IPM-EDTA DDST

The DDST using IPM and EDTA was employed to detect MBL production. Following CLSI standards, inocula were prepared from pure cultures of carbapenem-resistant *A. baumannii* (CRAB) as described above for antimicrobial susceptibility testing, adjusted to a 0.5 McFarland standard. A sterile, nontoxic cotton swab was dipped into the standardized inoculum, and excess fluid was expressed by rotating the swab firmly against the tube wall. The MHA plate was streaked three times at 60° angles to cover the entire surface.

**Figure 2:**
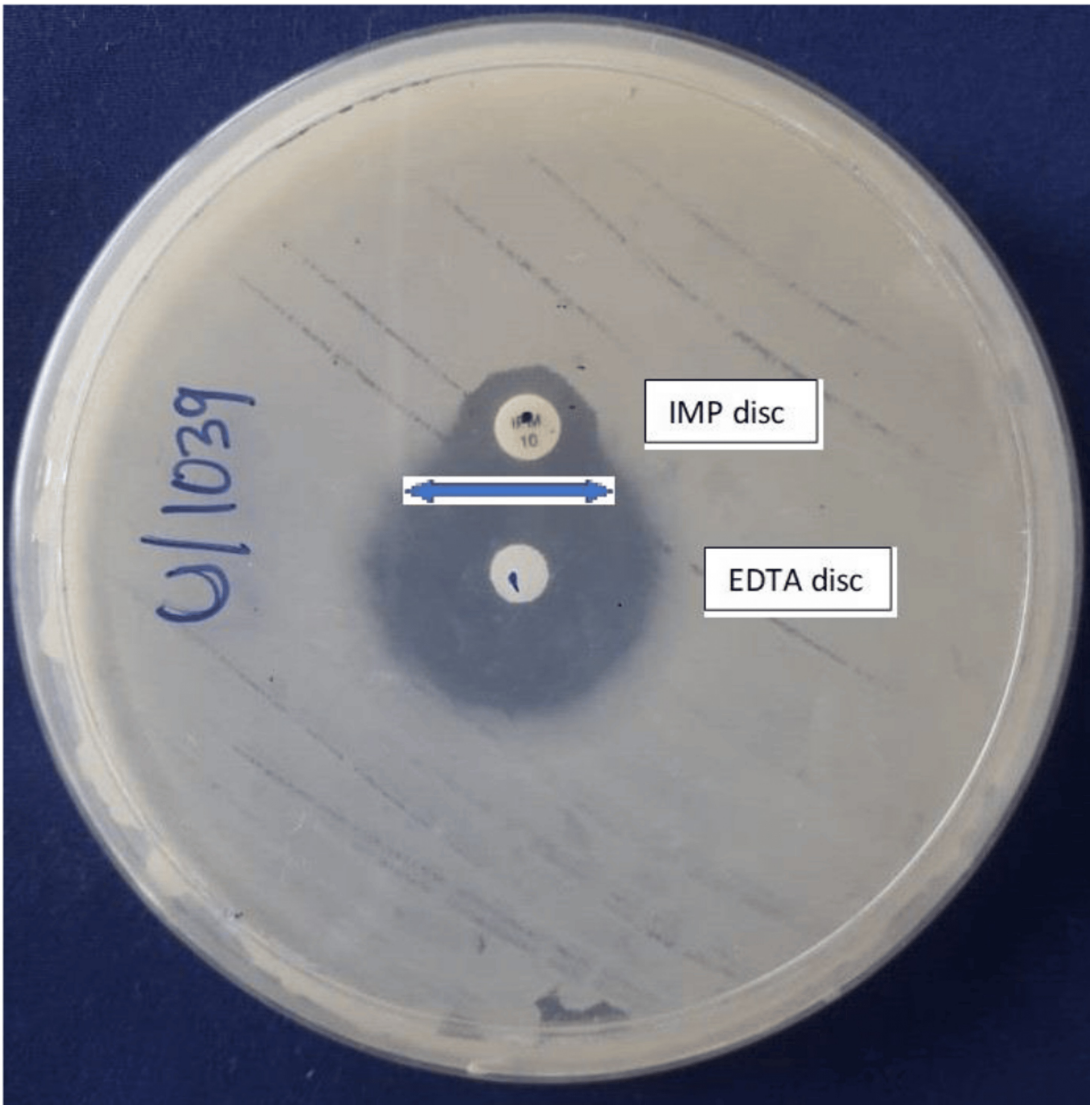
IPM-EDTA DDST for the detection of MBL producers The presence of an enlarged zone of inhibition toward the EDTA disc indicates positive MBL production, as shown in the figure. DDST, Double-Disk Synergy Test; IPM, imipenem; MBL, metallo-β-lactamase

After drying, a 10 µg IPM disc was placed on the agar, with a blank disc positioned 15 mm center-to-center from the IPM disc. Ten microliters of 0.5 M EDTA were applied to the blank disc using a micropipette and allowed to diffuse for a few minutes before overnight incubation of the plates. An enlarged zone of inhibition toward the EDTA disc was interpreted as positive for MBL production (Figure 2) [19].

MBL Epsilometer Test (E-Test)

The MBL E-test combines the principles of disc diffusion and MIC determination to detect both minimum inhibitory concentration (MIC) and MBL production. The test strip is coated with antibiotic at one end and antibiotic plus EDTA at the other. The concentration ranges used were IPM with EDTA, 1-64 μg/ml; IPM without EDTA, 4-256 μg/ml; MRP with EDTA, 1-64 μg/ml; and MRP without EDTA, 4-256 μg/ml.

**Figure 3:**
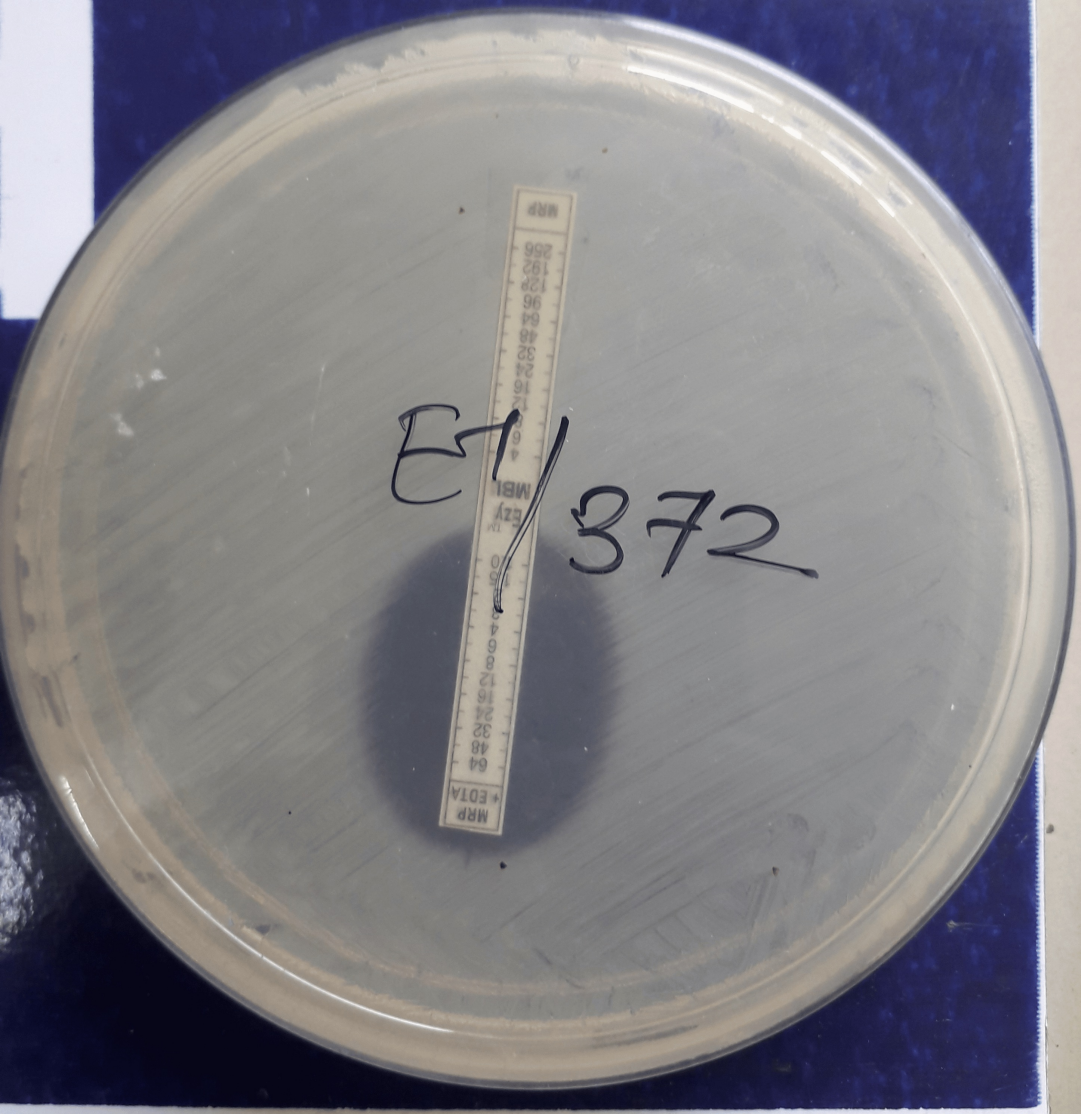
MBL E-test for the detection of MBL producers A reduction in MIC in the presence of MRP+EDTA by >8-fold indicates MBL activity, as shown in the figure. E-test, Epsilometer test; MBL, metallo-β-lactamase; MRP, meropenem

The test was performed on MHA, and results were read by viewing the strip from above the plate. A symmetrical inhibition ellipse formed, and the intersection of the ellipse with the E-test strip indicated the MIC value. MBL production was inferred if the MIC ratio between the two sides of the strip was ≥8, if a phantom zone appeared between IPM/IPM+EDTA, or if there was deformation of either ellipse (Figure 3) (MBL E-test technical manual, EM078 & EM092, HiMedia Laboratories Private Limited) [18].

Statistical analysis

Categorical variables were analyzed using frequency and percentage distributions, along with graphical representations. The sensitivity and specificity of the MHT and DDST were calculated using the MBL E-test as the gold standard for phenotypic detection of MBL production. Statistical analyses were performed using Microsoft Excel (Microsoft Corporation, Redmond, WA, USA).

## Results

A total of 168 *Acinetobacter* species were isolated from various clinical specimens, including *A. baumannii *(143; 85%), *Acinetobacter calcoaceticus* (12; 7%), *Acinetobacter lwoffii* (9; 5%), *Acinetobacter haemolyticus *(3; 2%), and *Acinetobacter junii *(1; 1%). Among the 143 *A. baumannii *isolates, 132 (92%) were resistant to carbapenems.

**Figure 4:**
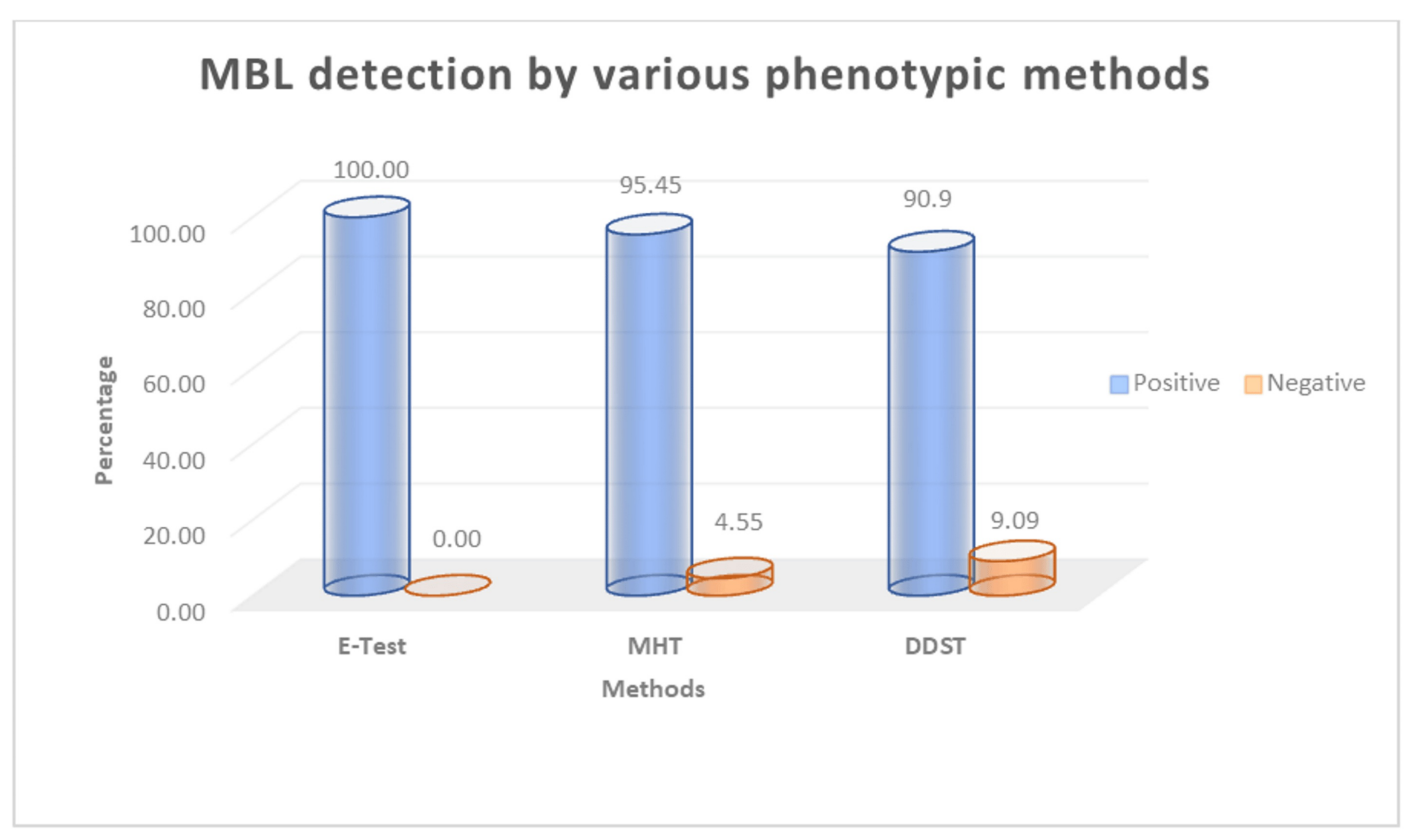
MBL detection in Acinetobacter baumannii by various phenotypic methods (n = 132) MBL, metallo-β-lactamase

Phenotypic confirmation of MBL production in CRAB was performed using the E-test, MHT, and DDST. Of the 132 CRAB isolates, 132 (100%) were positive by E-test, 126 (95.45%) were positive by MHT, and 120 (90.9%) were positive by DDST. The remaining 6 (4.54%) isolates were negative by MHT, and 12 (9.1%) were negative by DDST (Figure 4).

Using the E-test as the gold standard, the sensitivity and specificity of the other phenotypic tests were calculated. The MHT showed 95.45% sensitivity and 100.0% specificity (Table 1), while the DDST showed 90.91% sensitivity and 100.0% specificity (Table 2).

**Table 1:** Estimation sensitivity and specificity of MHT a = true positive; b = false positive; c = false negative; d = true negative E-test, Epsilometer test; MHT, Modified Hodge Test; NPV, negative predictive value; PPV, positive predictive value

MHT	E-test		
	Positive	Negative	Total
Positive	126	0	126
Negative	6	1	7
Total	132	1	133
Sensitivity (a/a + c)	0.95	95.45	-
Specificity (d/d + b)	1	100	-
PPV (a/a + b)	1	100	-
NPV (d/c + d)	0.14	14.29	-

**Table 2:** Estimation of sensitivity and specificity of the IPM-EDTA DDST a = true positive; b = false positive; c = false negative; d = true negative DDST, Double-Disk Synergy Test; E-test, Epsilometer test; IPM, imipenem; NPV, negative predictive value; PPV, positive predictive value

IPM-EDTA DDST	E-test		
	Positive	Negative	Total
Positive	120	0	120
Negative	12	1	13
Total	132	1	133
Sensitivity (a/a + c)	0.91	90.91	-
Specificity (d/d + b)	1	100	-
PPV (a/a + b)	1	100	-
NPV (d/c + d)	0.08	7.69	-

## Discussion

The World Health Organization has recognized CRAB as a “critical priority” pathogen for the development of novel antibiotics and anti-infective agents. Carbapenems are considered the last line of treatment for *A. baumannii*-associated infections, making the emergence of carbapenem resistance a major concern. Globally, there are increasing reports of carbapenem resistance, with studies in India suggesting that 40-75% of *A. baumannii* isolates are resistant to carbapenems. Such widespread resistance complicates empiric therapy, as isolates often display multidrug resistance to various antibacterial agents [7].

*A. baumannii* is often referred to as a “superbug” in hospital settings, particularly in intensive care units, due to its resistance to nearly all available antimicrobials [7]. The rise of antimicrobial resistance driven by MBL production is becoming increasingly prevalent. MBL-producing* A. baumannii *strains can cause severe infections and are easily transmitted via plasmids, facilitating rapid dissemination and adverse clinical outcomes. Early identification of MBL-producing isolates is therefore crucial to control the spread of resistance genes [1].

Phenotypic detection methods, such as the E-test, MHT, and DDST, are widely used for MBL detection in CRAB, while PCR remains the molecular gold standard. However, PCR is often impractical for routine diagnostic use due to its high cost and operational complexity [1,18,19]. In this study, phenotypic detection of MBL production in CRAB isolates was performed using the E-test, MHT, and DDST.

The MBL E-test is a quantitative method that determines the MIC while simultaneously detecting MBL production. By combining a β-lactam substrate with a β-lactamase inhibitor, the E-test identifies clinically significant MBLs, whether chromosomal or plasmid-mediated, in both aerobic and anaerobic bacteria. In our study, all 132 CRAB isolates (100%) tested positive using the E-test, consistent with previous studies by Shivaprasad et al. [18] (100%) and Vamsi et al. [13] (92%), but higher than the results reported by Buzila et al. [20] (79%) and Guzel et al. [21] (39%).

The MHT, originally developed by the CDC to detect carbapenemase in Enterobacteriaceae, is a simple screening method. While some isolates may produce false-positive results due to minor carbapenem hydrolysis by CTX-M or AmpC enzymes [18], our study demonstrated a high positivity rate of 95.45%, similar to Ranjbar and Farahani [22] (96%) and Anwar et al. [23] (83%), but higher than Guzel et al. [21] (21%), Buzila et al. [20] (42%), and Pattanaik et al. [24] (45%).

The DDST is another phenotypic method for detecting Ambler class B MBLs, with reported positivity rates ranging from 7% to 92% [13,16,18,23,25]. In our study, DDST detected 90.9% of CRAB isolates, consistent with Vamsi et al. [13] (92%) but higher than Shivaprasad et al. [18] (67%), Anwar et al. [23] (74%), Gupta et al. [16] (31%), and Purohit et al. [25] (7%).

Overall, all three phenotypic methods confirmed MBL production in clinical *A. baumannii* isolates, with the E-test demonstrating superior sensitivity and reliability. Based on sensitivity and specificity analyses, the MBL E-test is considered the most effective phenotypic method for detecting MBL-producing CRAB isolates.

## Conclusions

Routine screening for MBL-producing *A. baumannii *in diagnostic laboratories is crucial for guiding appropriate antimicrobial therapy and preventing nosocomial transmission. The simultaneous presence of multiple carbapenemases poses a challenge that must be carefully considered when implementing rigorous infection control protocols, continuous monitoring, and alternative or newer therapeutic approaches. Simple and reliable detection methods are needed for routine use in clinical laboratories. Among phenotypic methods, the MBL E-test has demonstrated superior diagnostic performance, although molecular confirmation remains the gold standard.

## References

[1] Kulkarni SS, Mulay MV (2022). Phenotypic detection of metallo-beta-lactamase production in clinical isolates of Escherichia coli and Klebsiella pneumoniae in a tertiary care hospital. MGM J Med Sci.

[2] Pfeifer Y, Cullik A, Witte W (2010). Resistance to cephalosporins and carbapenems in Gram-negative bacterial pathogens. Int J Med Microbiol.

[3] Öztürk H, Ozkirimli E, Özgür A (2015). Classification of beta-lactamases and penicillin binding proteins using ligand-centric network models. PLoS ONE.

[4] Evans BA, Hamouda A, Amyes SG (2013). The rise of carbapenem-resistant Acinetobacter baumannii. Curr Pharm Des.

[5] Javkar K, Rand H, Hoffmann M (2021). Whole-genome assessment of clinical acinetobacter baumannii isolates uncovers potentially novel factors influencing carbapenem resistance. Front Microbiol.

[6] Vijayakumar S, Gopi R, Gunasekaran P, Bharathy M, Walia K, Anandan S, Veeraraghavan B (2016). Molecular characterization of invasive carbapenem-resistant Acinetobacter baumannii from a tertiary care hospital in South India. Infect Dis Ther.

[7] Vijayakumar S, Mathur P, Kapil A (2019). Molecular characterization & epidemiology of carbapenem-resistant Acinetobacter baumannii collected across India. Indian J Med Res.

[8] Butler DA, Biagi M, Tan X, Qasmieh S, Bulman ZP, Wenzler E (2019). Multidrug resistant Acinetobacter baumannii: resistance by any other name would still be hard to treat. Curr Infect Dis Rep.

[9] Poirel L, Bonnin RA, Nordmann P (2011). Genetic basis of antibiotic resistance in pathogenic Acinetobacter species. IUBMB Life.

[10] Tripathi P, Gajbhiye S (2013). Prevalence of multidrug resistance, ESBL and MBL production in Acinetobacter spp. Int J Recent Trends Sci Technol.

[11] Bush K (2001). New β-lactamases in gram-negative bacteria: diversity and impact on the selection of antimicrobial therapy. Clin Infect Dis.

[12] Hughes AJ, Ariffin N, Huat TL (2005). Prevalence of nosocomial infection and antibiotic use at a university medical center in Malaysia. Infect Control Hosp Epidemiol.

[13] Vamsi KS, Moorthy SR, Murali TS (2021). Phenotypic methods for the detection of metallo-beta-lactamase production by gram-negative bacterial isolates from hospitalized patients in a tertiary care hospital in India. J Pure Appl Microbiol.

[14] Collee JG, Miles RS, Watt B (1996). Tests for the identification of bacteria. Mackie and McCartney Practical Medical Microbiology, 14th Edition.

[15] Procop GW, Koneman EW (2017). Koneman Diagnostic Microbiology, 7th Edition. Koneman's Color Atlas and Textbook of Diagnostic Microbiology. 7th ed. United States: Wolters Kluwer Health.

[16] Gupta N, Gandham N, Jadhav S, Mishra RN (2015). Isolation and identification of Acinetobacter species with special reference to antibiotic resistance. J Nat Sci Biol Med.

[17] (2019). CLSI M100: Performance Standards for Antimicrobial Susceptibility Testing, 29th Edition. Wayne, PA: Clinical and Laboratory Standards Institute.

[18] Shivaprasad A, Antony B, Shenoy P (2014). Comparative evaluation of four phenotypic tests for detection of metallo-β-lactamase and carbapenemase production in Acinetobacter baumannii. J Clin Diagn Res.

[19] Lee K, Lim YS, Yong D, Yum JH, Chong Y (2003). Evaluation of the Hodge test and the imipenem-EDTA double-disk synergy test for differentiating metallo-β-lactamase-producing isolates of Pseudomonas spp. and Acinetobacter spp. J Clin Microbiol.

[20] Buzila ER, Dorneanu OS, Luncă C, Jelihovschi I, Iancu LS (2022). Phenotypic carbapenemase production and blaOXA detecting by PCR in Acinetobacter baumannii isolates from a hospital of infectious diseases from North-East Romania. Rev Rom Med Lab.

[21] Guzel M, Afsar Y, Akdogan D, Moncheva P, Hristova P, Erdem G (2018). Evaluation of metallo-beta-lactamase production in multiple antibiotic-resistant Pseudomonas spp. and Acinetobacter baumannii strains. Biotechnol Biotechnol Equip.

[22] Ranjbar R, Farahani A (2019). Study of genetic diversity, biofilm formation, and detection of Carbapenemase, MBL, ESBL, and tetracycline resistance genes in multidrug-resistant Acinetobacter baumannii isolated from burn wound infections in Iran. Antimicrob Resist Infect Control.

[23] Anwar M, Ejaz H, Zafar A, Hamid H (2016). Phenotypic detection of metallo-beta-lactamases in carbapenem resistant Acinetobacter baumannii isolated from pediatric patients in Pakistan. J Pathog.

[24] Pattanaik A, Banashankari GS (2019). Characterisation of Acinetobacter with special reference to carbapenem resistance and biofilm formation. Trop J Path Micro.

[25] Purohit M, Mendiratta DK, Deotale VS, Madhan M, Manoharan A, Narang P (2012). Detection of metallo-β-lactamases producing Acinetobacter baumannii using microbiological assay, disc synergy test and PCR. Indian J Med Microbiol.

